# Rebound From Restoration: Assessing the Diversity of Bird Assemblages in Revegetated and Remnant Patches of Critically Endangered Lowland Subtropical Rainforest

**DOI:** 10.1002/ece3.72345

**Published:** 2025-10-17

**Authors:** Marley Borrow, Susan Fuller, Brendan Doohan

**Affiliations:** ^1^ School of Biology and Environmental Science Queensland University of Technology Brisbane Australia

**Keywords:** functional diversity, landscape connectivity, passive acoustic monitoring, phylogenetic diversity, species richness estimators

## Abstract

With increased rates of biodiversity loss and deforestation, ecological restoration has become a common practice worldwide. Despite this, metrics that are often used to quantify the success of biodiversity recovery display highly variable patterns depending upon the ecosystem being restored. The aims of this study were to investigate four biodiversity metrics – Chao2 species richness, rainforest‐dependent species richness, functional diversity and phylogenetic diversity – of bird assemblages occurring in connected remnant, fragmented remnant, old revegetation and young revegetation patches of critically endangered Australian lowland subtropical rainforest (LSR). The study also examined the influence of site‐based vegetation and landscape attributes on bird assemblages in these patches. A combination of passive acoustic monitoring and bird surveys revealed 72 species across eight study sites. While no site‐based vegetation attributes were significant predictors of avian diversity, high canopy cover had a strong positive relationship with rainforest‐dependent species. The landscape context surrounding the patches had a significant relationship with three metrics, with vegetated vs. cleared land within a 200 and 500 m radius from the recording location being a significant positive predictor of species richness, rainforest‐dependent species richness and functional diversity. A non‐metric multidimensional scaling analysis showed that rainforest‐dependent species were associated with sites connected to uncleared remnant vegetation in the broader landscape, while generalist species were associated with fragmented sites. The results of our study highlight the importance of ecological restoration for the recovery of bird assemblages in Australian LSR, as well as the variables that may influence fluctuations in species richness, rainforest‐dependent species richness, functional diversity and phylogenetic diversity within these communities. Future ecological restoration initiatives should aim to re‐establish canopy cover and restore the connectivity and extent of LSR to facilitate the recolonisation of diverse bird assemblages that depend on this critically endangered ecosystem.

## Graphical abstract

We investigated the diversity and composition of bird assemblages in critically endangered lowland subtropical rainforest (LSR) in northern New South Wales, Australia. We used traditional bird surveys, passive acoustic monitoring and artificial intelligence to investigate the diversity and composition of bird assemblages in restored and remnant patches of LSR. We conclude that ecological restoration initiatives that re‐establish canopy cover and restore the connectivity and extent of LSR facilitate the recolonisation of diverse bird assemblages that depend on this critically endangered ecosystem.

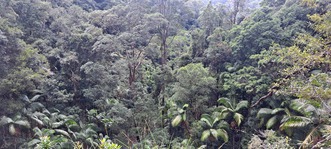

## 1 Introduction

Biodiversity loss is widely recognised as a significant environmental issue in the Anthropocene (Betts et al. 2017; Steffen et al. 2015). Since 1500 AD, an estimated 16 to 50% of global species have become threatened or gone extinct, primarily due to changes in land and sea use, overexploitation of ecosystem resources, climate change, pollution and the introduction of invasive species (Isbell et al. 2023; Jaureguiberry et al. 2022; Palombo 2021; Maxwell et al. 2016; Steffen et al. 2015; Bradshaw 2012). Australia has consistently exhibited one of the highest rates of biodiversity loss worldwide, with at least 316 bird species becoming threatened or extinct since historical records began (Garnett and Baker 2021). Since European settlement, deforestation has been a key driver of biodiversity loss in Australia, with ~48% of the continent's land cover significantly altered for agriculture, urban development and logging (Evans 2016; Laurance et al. 2014; Bradshaw 2012; Munro et al. 2007; Lindenmayer et al. 2000). Today, much of Australia's remaining old‐growth forest has become heavily fragmented, resulting in poor connectivity across the landscape (Parkes et al. 2012; Lindenmayer et al. 2000). Such fragmentation has severe impacts on native taxa, which are reliant on old‐growth vegetation for food sources, breeding, dispersal and protection from predation (McAlpine et al. 2002). Fragmentation can lead to reduced genetic diversity, intensified competition for resources and increased risk of disease and predation (Schlaepfer et al. 2018; May and Norton 1996).

One such ecosystem that has experienced significant deforestation due to urbanisation and agricultural expansion is the lowland subtropical rainforest community (henceforth LSR) in Australia (Parkes et al. 2012; Erskine et al. 2007; Lymburner et al. 2006; Catterall et al. 2004; Floyd 1990). Located in the subtropical coastal regions of eastern Australia between the latitudes of 25.53° S and 32.93° S, the LSR spanned over an estimated 196,110 ha prior to European settlement. However, the ecosystem's spatial extent has declined over 90% and is classified as critically endangered under the Australian Federal Environmental Protection and Biodiversity Act 1999 (Commonwealth of Australia 1999). Lowland subtropical rainforest exhibits an extensive array of biodiversity, harbouring ancient avian lineages that date back to the supercontinent Gondwana (e.g., Albert's lyrebird, Australian logrunner, marbled frogmouth; Mitchell et al. 2021). However, intensive clearing and fragmentation of LSR have had significant consequences on its biodiversity, as the small patches that remain do not provide sufficient habitat and functional niches to support LSR‐associated species assemblages. Such fragmentation exposes populations to complications of reduced genetic variability, population fitness and poor adaptation to further climatic and anthropogenic pressures (Kooyman et al. 2012; Vetter et al. 2011; Rossetto et al. 2004). To rectify this issue, ecological restoration has become the dominant method to protect and restore LSR communities; the primary focus being to connect and expand the geographic extent of existing LSR, as well as maintaining revegetated fragments so they reach a mature, self‐sustaining state (Parkes et al. 2012; Catterall and Harrison 2006; Lymburner et al. 2006).

Ecological restoration is a process that aims to assist in the recovery of an ecosystem which has been degraded, damaged or destroyed (Gann et al. 2019). Through active management interventions, such as the reintroduction of plants and wildlife, removal of dams and soil amendments, the structure, function and biodiversity of degraded ecosystems may return to levels that resemble pre‐disturbance ecosystems (Martin 2017). Ecological restoration of forest communities typically requires either large‐scale planting initiatives (often implemented in severely disturbed landscapes to establish or reconnect vegetation cover that has been cleared or fragmented for substantial periods of time) or assist the natural regenerative processes of a pre‐dispersed seedbank to expand and diversify an existing vegetation patch (Campbell et al. 2017; Uebel et al. 2017; Guidetti et al. 2016; Munro et al. 2012). Through restoring native vegetation cover and diversity, it is assumed that mobile species associated with these habitats will passively return over time (known as the ‘Field of Dreams’ hypothesis, Palmer et al. 1997). However, it is possible for restored patches to mature into alternative states unsuitable for the target species, or species to fail to immigrate from surrounding patches (Munro et al. 2007; Catterall et al. 2004; Kanowski et al. 2003). As such, restoration projects must be monitored to ensure they reach their targeted outcomes.

Metrics are required to measure ecological restoration success, including those that provide an estimate of species diversity or act as a surrogate for ecosystem function (e.g., functional diversity), as well as the genetic differentiation of species (e.g., phylogenetic diversity). To obtain measurable differences in these metrics, biodiversity monitoring should be undertaken at regular intervals (Prach et al. 2019; Lindenmayer et al. 2012; Green et al. 2005). Traditional methods of biodiversity monitoring involve intensive and expensive manual collection of data (i.e., wildlife trapping, on‐ground fauna and flora surveys). Recent technological advancements have led to the development of a range of remote monitoring techniques (i.e., passive acoustic monitoring (PAM), motion‐detection cameras, etc.) and machine learning methods (i.e., recognition software) that allow more efficient surveys to be conducted without surveyors being present on site. Passive acoustic monitoring can be implemented over large spatial scales and continuous recording periods to provide valuable data on soniferous biodiversity and is comparatively non‐invasive relative to traditional observer‐based surveys (Browning et al. 2017). This technology is particularly beneficial for monitoring birds, as they are often highly mobile, are (mostly) vocal and easy to survey and are found in a wide variety of ecosystems (Fraixedas et al. 2020; Blair 1999).

### 1.1 Study Aims

Ecological restoration initiatives undertaken in Australian LSR patches have led to an increase in the spatial extent of LSR vegetation; however, the success of these practices in restoring faunal biodiversity in revegetated patches remains largely anecdotal. To address this knowledge gap, birds were selected as restoration indicator taxa, due to the taxonomic and functional diversity of avian assemblages known to occur in LSR, as well as their sensitivity to habitat loss and degradation (Pavlacky Jr et al. 2015; Catterall et al. 2012). Furthermore, birds can be remotely surveyed using PAM, and machine learning methods have been developed to efficiently detect multiple species in large acoustic datasets (Kahl et al. 2021). Therefore, the aim of this study was to characterise avian biodiversity using four metrics – species richness, rainforest‐dependent species richness, functional diversity and phylogenetic diversity – with the goal of identifying vegetation and landscape features that influence the recovery of bird assemblages in restored and remnant patches of critically endangered Australian LSR.

## 2 Methods

### 2.1 Study Sites

The study was conducted in the Big Scrub LSR (henceforth Big Scrub) found in northern New South Wales, eastern Australia. The region experiences a humid subtropical climate, with annual precipitation averaging 1340 mL and average summer temperatures ranging between 20°C and 35°C (Bureau of Meteorology 2025). The altitude of the study region is typically below 250 m above sea level. Once covering 75,000 ha of continuous LSR, it has been intensively cleared for agricultural and urban development since European settlement (Floyd 1990). This has resulted in a 99% reduction in the Big Scrub's geographic range, with the remaining remnants of this vegetation occurring today as isolated fragments across the Big Scrub's original extent. However, in the past 30 years, ecological restoration initiatives have attempted to restore LSR vegetation, with approximately 650 ha being actively converted from open pasture to secondary‐phase rainforest (T. Parkes, personal communication). The systematic control of weed species has also resulted in accelerated recruitment of a range of LSR plant species, with approximately 250 ha treated via mechanical weeding and herbicide annually across 38 remnant patches (T. Parkes, personal communication).

**FIGURE 1:**
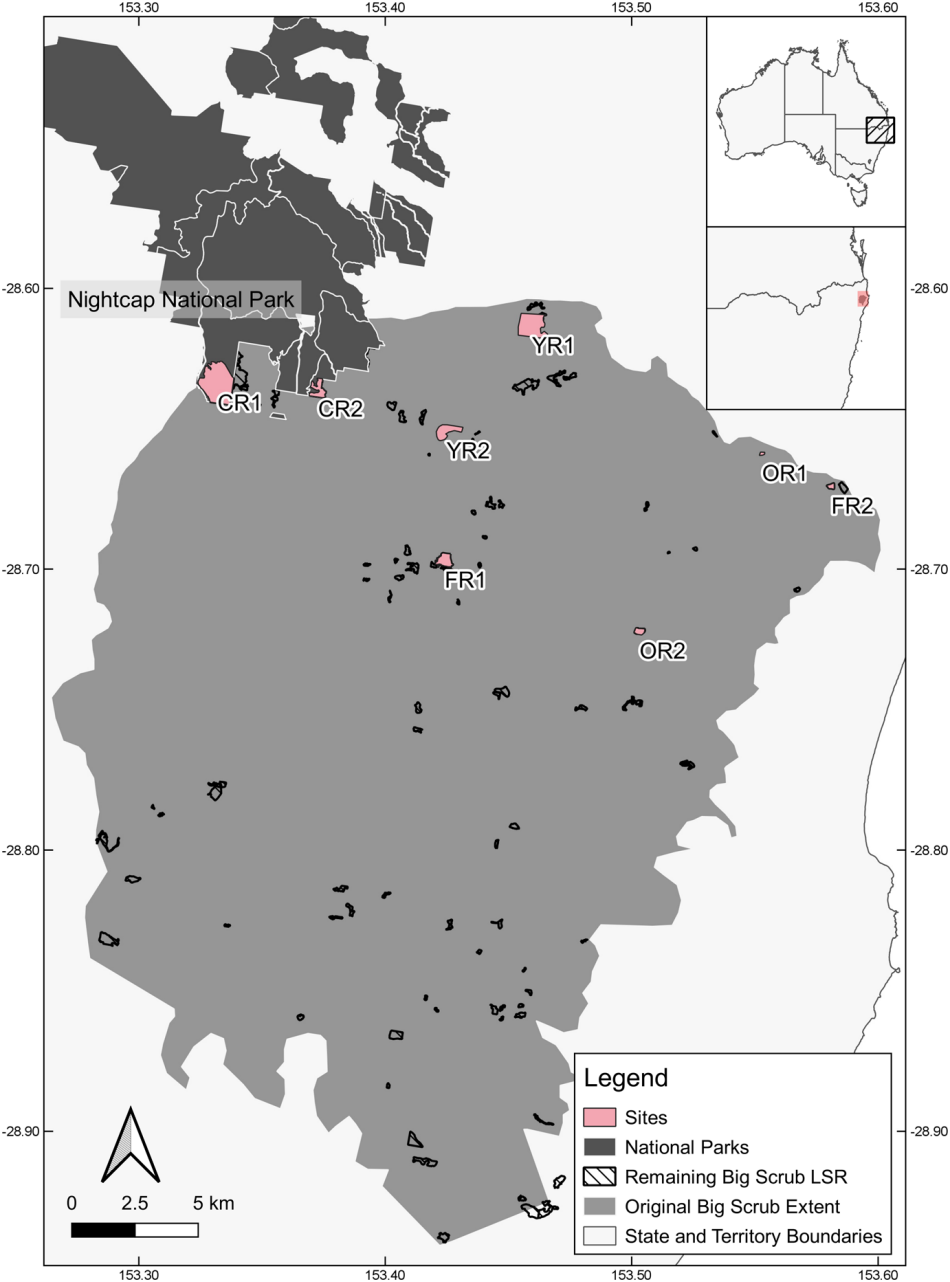
Location of the eight study sites in relation to the original extent of the Big Scrub Lowland Subtropical Rainforest (light grey). Remnant Big Scrub LSR patches shown in black and white hatched lines. National parks abutting remnants shown in dark grey. Site categories: Connected Remnant (CR), Fragmented Remnant (FR), Old Revegetation (OR), Young Revegetation (YR). Sites in order are Big Scrub Flora Reserve (CR1), Boomerang Falls (CR2), Andrew Johnston's Big Scrub (FR1), Hayter's Hill West (FR2), Brookfarm (OR1), Tarra (OR2), Emerald Valley (YR2) and Lune de Sang (YR2). Inset maps show study location in New South Wales, Australia.

Sites were selected within the northern distribution of the Big Scrub's previous extent so that climatic conditions were consistent. Sites were required to consist of > 70% LSR coverage within their vegetation boundaries (as per aerial imagery; NSW Department of Climate Change, Energy, the Environment and Water 2025). Four categories were chosen to capture variation in restoration age and connectivity across sites. These included connected remnant (uncleared vegetation with high connectivity to large remnant LSR patches; e.g., abutting national parks), fragmented remnant (uncleared vegetation with low connectivity to large remnant LSR patches; e.g., predominantly surrounded by cleared or agricultural land), old revegetation (previously cleared vegetation with 25–30 years of regeneration) and young revegetation (previously cleared vegetation with 5–15 years of regeneration). Given the extent of historic clearing of this vegetation community, and the existence of only two connected remnant sites, two sites were selected in each of these four categories, resulting in a total of eight sites surveyed (Figure 1). Survey sites were an average of 4.5 km (range 3 to 8.5 km) from the next closest survey site. Survey site patches had an average area of 45.3 ha (min 2.1 ha, max 168.9 ha).

### 2.2 Passive Acoustic Monitoring

At each site, one BAR‐LT acoustic recorder (Frontier Labs) was deployed two metres above the ground via attachment to a star picket. Recorders were at least 200 m from site edges to reduce the impact of edge effects (Neate‐Clegg et al. 2016). BAR‐LT units were scheduled to record at a sampling rate of 22 kHz for 2 h at dawn and dusk each day. An additional 10‐min block was recorded 3 h post dawn chorus and 4 h post dusk chorus. Site coordinates were incorporated into recording schedules to allow for fluctuations in temporal changes in sunrise and sunset over the calendar year. Recorders were deployed for 94 days (14th March–16th June 2024).

Twenty days were randomly selected from the recordings for analysis. Any days that experienced ‘interference’ (i.e., rain, wind and machinery) were removed and supplemented with the closest day in which a disturbance did not occur. Species accumulation curves, calculated using Chao2 species richness estimators and a 95% confidence interval, were used to justify the 20‐day sample period (Chao and Chiu 2016). A combination of manual annotation and BirdNET (Version 1.3.1; Kahl et al. 2024) was used to detect species presence/absence across sites. BirdNET annotations were generated using the geographic coordinates of recording locations to refine the recogniser species lists (Kahl et al. 2024). A 0.1 confidence score was used to maximise detections due to the limited training data available for Australian LSR bird species. BirdNET annotations were output as a Raven selection table and manually verified in Raven Pro (Version 1.6) to ensure the validity of species detections. Species detections were also cross‐referenced with Fitzgerald et al. (2021) and Holmes (1987) to confirm recent and historical records of identified bird species occurring within the Big Scrub LSR. Once a detection was confirmed, it was compiled into a site‐specific species list. The Working List of Australian Birds nomenclature was used for all species names in this study.

### 2.3 Point Count Surveys

To visually confirm the presence of bird species, as well as to increase the chances of detecting cryptic or non‐vocal species, a single *ad‐hoc* standard point count bird survey with a fixed radius was undertaken at each recording site. Surveys were conducted at dawn by the same observer (MB), with the recording device location as the center of the radius. Each survey ran for 20 min, with the species name, method of detection (seen, heard, or seen + heard) and the vegetation layer (canopy, mid story, ground) in which they were detected recorded. Species identified within or above the vegetation patch's boundaries were also documented but were noted as incidental observations. Results from the point count surveys and survey effort for each site were included in the species lists.

### 2.4 Vegetation Attributes

Vegetation surveys were conducted at each site to quantify six vegetation attributes. A 50 m line transect was centred around the recording device and positioned along the prevailing topographic contour. Cover of canopy vegetation, mid‐story vegetation and non‐native species was calculated by summing the total metres these vegetation categories were positioned directly above the transect line (Eyre et al. 2015). A 25 m × 10 m plot was positioned around the 50 m line transect (mid‐line of the plot), and the height and diameter at breast height (DBH) of five randomly selected trees and shrubs were measured. The sum of the length of coarse woody debris (> 10 mm diameter) occurring in the ground layer of the 25 m × 10 m plot was also recorded. Five 1 m × 1 m quadrats were placed along the 50 m mid‐line, and the cover of native forbs, shrubs, grasses, organic leaf litter and non‐native forbs/grasses were estimated to the nearest 5%. Any fruiting or flowering plant species in the LSR patch were recorded as an incidental observation.

### 2.5 Landscape Attributes

Remotely sensed data were used to categorise and calculate landscape metrics. To determine the vegetation communities around the sensors, the New South Wales Plant Community Types (PCT) (2023) and CRAFTI Upper North‐East Floristics VIS 1108 (2012) spatial layers were acquired from the New South Wales BioNet Vegetation Classification database and cross referenced with sensor GPS coordinates. Four landscape attributes associated with patch size, connectivity and isolation (Adler and Jedicke 2022) were calculated at each site. These included the total vegetated area within the sites' boundaries, percentage of native vegetation cover relative to cleared area (i.e., grazing and urban land uses), percentage of native vegetation relative to non‐native vegetation and percentage of LSR relative to other vegetation (native + non‐native). All landscape attributes were calculated within a 100, 200 and 500 m radius buffer surrounding the acoustic sensor using ArcGIS Pro (version 2.8; Esri 2021).

### 2.6 Biodiversity Metrics

Species richness (total number of unique species detected at the site over the course of the study) was tallied at each site, and a Chao2 species richness estimate was calculated to account for possible undetected species. While species richness is commonly used in many studies, many non‐rainforest‐dependent bird species inhabit woodlands and cleared areas surrounding Big Scrub LSR and may utilise these patches regardless of vegetative attributes. This has the potential to mask trends in biodiversity recovery (Doohan et al. 2019). Therefore, rainforest‐dependent species richness was determined based on species descriptions in the Handbook of Australian, New Zealand and Antarctic Birds (HANZAB; BirdLife Australia 2023).

A functional diversity matrix was produced using site‐specific species lists and the following functional group traits: feeding guild, foraging height, time of most foraging activity (diurnal/nocturnal) and species body mass. This trait information was compiled using the Wilman et al. (2014) dataset. Feeding guilds were allocated to species based on their primary food type (i.e., insectivore, frugivore, herbivore, nectarivore, granivore, carnivore), with omnivorous species being categorised in the group of their most favourable food when a variety of feeding sources were available. The foraging height of species was determined as the highest stratum at which a species most commonly occurs (Doohan et al. 2019; Menkhorst et al. 2017). Foraging heights were scaled in value between 1 and 4. The value of 1 was assigned to ground foraging species, 2 to species which forage in the mid story, 3 to species which forage in the canopy, and 4 was assigned to aerial foraging species. Petchey's FD was then calculated as this metric incorporates multiple functional traits of species (Petchey and Gaston 2002) and has been found to be informative in other avian assemblage studies (Doohan et al. 2019). As the body mass of species in this study was heavily left skewed, species body mass was log10 transformed. Custom code in R statistical software (Version 4.2.0) was used to generate a Gower's distance matrix and to calculate FD (see Figshare repository).

Phylogenetic diversity was measured as the mean phylogenetic distance of site‐specific bird assemblages. The phylogenetic tree of birds from Jetz et al. (2012) was used; however, due to the age of the publication, the scientific names of eight species were updated to their current classifications for the analysis (Appendix S1). Phylogenetic trees were constructed for each site based on the presence/absence species data for each site. Trees were pruned in the R statistical environment using the ‘U.Phylomaker’ package (Jin and Qian 2023), and the ‘ape’ package (Paradis and Schliep 2019) was used to calculate the mean cophenetic distance of species across sites.

### 2.7 Statistical Analysis

Due to the low number of replicates in each site category (*n* = 2), descriptive statistics implemented in R (R Core Team 2024) were used to examine differences between categories to avoid type II errors. To examine the relationships between diversity metrics and site‐based vegetation attributes and landscape metrics, a univariate regression screening approach was used. Each diversity metric was separately tested against each vegetation and landscape variable, and a linear regression model was fitted to estimate the slope (effect size). To account for non‐normal data distributions and to avoid type I error, permutation tests from the R package Coin (Hothorn et al. 2008) were used to generate 9999 re‐samplings of the data so that statistical significance could be assessed. To ensure confidence intervals were robust, 1000 bootstrap resampling was used to generate percentile confidence intervals for the regression slopes. A non‐metric multidimensional scaling analysis (NDMS) was undertaken using the ‘metaMDS’ function in the ‘vegan’ R package (Dixon 2003) to visually explore species associations across sites. The R package ‘ggrepel’ (Slowikowski 2025) was used to offset overlapping species names in the plot. Potential species associations were determined by their unique occurrence at one or both sites within a site category (e.g., Connected remnant site 1 and Connected remnant site 2). Species that were found in more than one category were deemed ‘not associated’.

## 3 Results

### 3.1 Biodiversity Metrics

A total of 72 species were recorded across all sites (Appendix S2). The Chao2 95% confidence intervals overlapped the species richness estimates at all eight sites (Table 1), indicating that the 20‐day sampling period of acoustic recordings was sufficient to detect most species present at the sites. However, it should be noted that the Chao2 95% confidence intervals for Boomerang Falls (Connected remnant site 2) and Andrew Johnstons' Big Scrub (Fragmented remnant site 1) were considerably wider than the species richness estimates, indicating species detections may increase with a longer sampling period. Of the 72 species detected over the duration of the study, 15 were identified as rainforest‐dependent (Appendix S2).

**TABLE 1:** Diversity metrics (Chao2 species richness, rainforest‐dependent, functional diversity, phylogenetic diversity) for each site.

Site name	Site code	Chao2 SR (95% confidence interval)	RD	Petchey's FD	PD
Big Scrub Flora Reserve	CR1	52.27 (48.79–55.75)	12	6.92	144.26
Boomerang Falls	CR2	62.62 (40.82–84.42)	15	7.14	145.32
Andrew Johnston's Big Scrub	FR1	53.62 (31.82–75.42)	11	7.18	148.59
Hayter's Hill West	FR2	40.14 (33.77–46.51)	6	6.06	137.66
Brookfarm	OR1	38.48 (35.99–40.96)	7	7.12	140.43
Tarra	OR2	44.48 (42.53–46.42)	5	6.56	143.60
Emerald Valley	YR1	52.81 (42.90–62.72)	7	6.77	137.49
Lune de Sang	YR2	54.81 (44.90–64.72)	8	6.80	142.86

*Note:* Site codes indicate site category: connected remnant (CR), fragmented remnant (FR), old revegetation (OR), young revegetation (YR).

A total of 32 bird species found across all sites were insectivores, 15 frugivores, 10 carnivores, 6 granivores, 5 herbivores and 4 nectivores (Appendix S3). There were 37 canopy foraging species, while 16 foraged in the mid‐story and 10 occurred as ground foraging specialists. Sixty‐six species were classed as predominantly diurnal, while six were identified as nocturnal (Appendix S3). Most species scored between 2.0 and 2.5 for log_10_ mass (*n* = 20), while only a few species scored between 3.5 and 4.0 (*n* = 2).

Connected remnant sites had the highest values for all four metrics, with Boomerang Falls (connected remnant site 2) having the highest Chao2 species richness and rainforest‐dependent species richness values across all sites (Table 1). The two fragmented remnant sites displayed contrasting results (Table 1), with Andrew Johnstons Big Scrub (fragmented remnant site 1) exhibiting similar values to the connected remnant sites, while Hayter's Hill West (fragmented remnant site 2) had low values for all four metrics relative to other sites. Notably, Andrew Johnstons Big Scrub had the highest phylogenetic diversity of all sites due to the high number (29) of taxonomic families found at the site. Old revegetation had generally low values for all metrics. The young revegetation produced mixed results with high Chao2 species richness values, but low–mid rainforest‐dependent species richness, functional diversity and phylogenetic diversity values.

### 3.2 Vegetation and Landscape Attribute Analyses

Despite the differences in the ages of remnant and revegetation sites, vegetation metrics were similar between site categories (Appendix S4 and Figure S1). This was particularly the case for ground cover and understory vegetation where in some cases young revegetation had higher values than connected remnant (mid story cover mean 91% and 74% respectively). Canopy cover was the only metric that showed a consistent pattern, with connected remnant having the highest canopy cover (mean 92%), followed by fragmented remnant and old revegetation (both mean 85%) and new revegetation (mean 77%). Structural elements of the canopy (canopy cover, height and width) were low in new revegetation sites and were all substantially lower than connected remnant sites. Despite this, both old revegetation and fragmented vegetation had similar canopy height and width to connected remnant sites. The results of our regressions showed no significant relationships between site‐based vegetation attributes and any diversity metric (Appendix S5). A near significant relationship was detected between rainforest‐dependent species and percentage canopy cover (37.86 CI [−9.01–99.33] *p* = 0.08) (Appendix S5).

**FIGURE 2:**
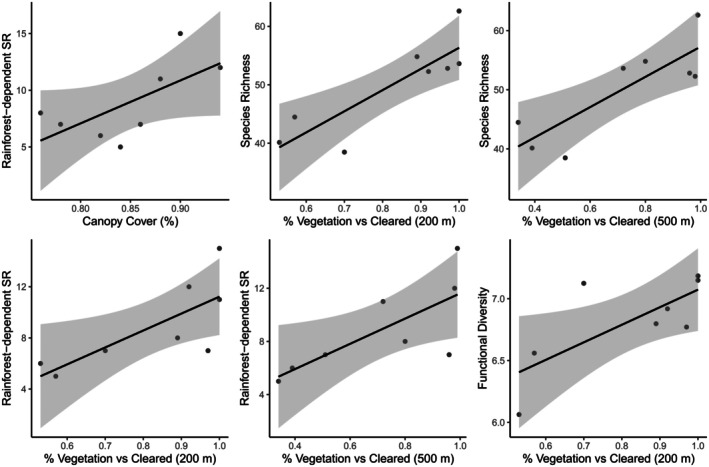
Visualisation of significant regression models for Chao2 species richness, rainforest‐dependent species richness and functional diversity in relation to percentage of vegetated landscape within a 200 and 500 m radius of the sensors (line of best fit in black and grey shading indicates confidence intervals).

Chao2 species richness, rainforest‐dependent species richness and functional diversity exhibited significant positive relationships with the percentage of vegetated landscape within a 200 and 500 m radius of the sensors (Figure 2). The strongest relationships were found for Chao2 species richness (slope = 36.22, CI [22.08, 65.35], *p* = 0.008), rainforest‐dependent species richness (slope = 13.23, CI [4.42, 24.28], *p* = 0.03) and functional diversity (slope = 1.42, CI [−0.35, 2.40], *p* = 0.03) at a 200 m radius, while both species richness and rainforest‐dependent species richness had a significant positive relationship at a 500 m radius (species richness slope = 25.58, CI [10.61, 41.59], *p* = 0.01; rainforest‐dependent species richness slope = 9.5, CI [2.42, 15.36], *p* = 0.04). All other variables did not yield any significant relationships (Appendix S5). There were no significant relationships between landscape attributes and phylogenetic diversity.

### 3.3 Bird Assemblages

**FIGURE 3:**
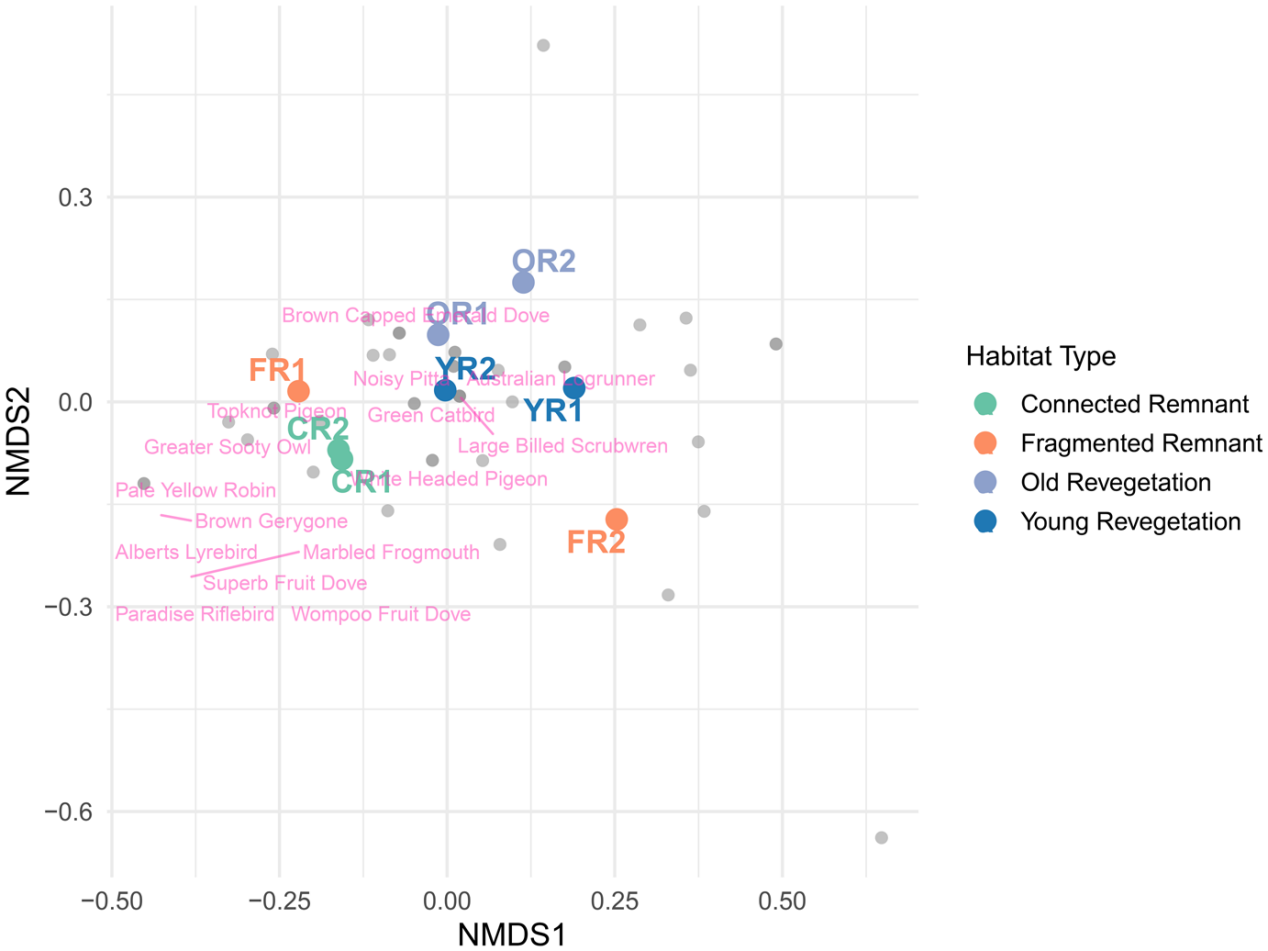
NMDS of species composition across sites and site categories. NMDS stress = 0.06. Green = connected remnant (CR), orange = fragmented remnant (FR), light blue = old revegetation (OR), dark blue = young revegetation (YR). Rainforest‐dependent species are indicated by pink text. Non‐rainforest‐dependent species are represented by unlabelled grey points.

Results from the NMDS analysis revealed close clustering of the two connected remnant sites, indicating very similar bird species assemblages (Figure 3). The young revegetation sites did not cluster as closely but were still adjacent to each other in the NMDS, indicating some sharing of species between the two sites, and a similar pattern was found for the old revegetation sites (Figure 3). In contrast, the fragmented remnant sites were disassociated from each other, with fragmented remnant site 1 (Andrew Johnston's Big Scrub) clustering with the connected remnant sites, while fragmented site 2 (Hayter's Hill) was distant in the NMDS, indicating that many species were different at this site relative to all other sites (Figure 3). Rainforest‐dependent species were more associated with fragmented remnant 1 and the two connected remnant sites in ordinal space.

## 4 Discussion

This study investigated the diversity and composition of bird assemblages at eight sites in remnant and revegetated Big Scrub LSR in northern New South Wales, Australia. The aims of this study were to identify the contribution that restoration initiatives have to the recovery of bird assemblages in critically endangered LSR, as well as the influential factors that may affect the composition of these assemblages. Our results show that all four metrics examined in this study (Chao2 species richness, rainforest‐dependent species richness, functional diversity and phylogenetic diversity) had highest values in fragmented remnant 1 and the two connected remnant sites and were generally lower in the revegetated sites. Nevertheless, many bird species were detected in the revegetated LSR patches surveyed in this study, particularly in young restoration. The relationships between diversity metrics and site‐based vegetation characteristics revealed that percentage canopy cover was an important determinant of the presence of rainforest‐dependent species, and that the proportion of vegetated vs. cleared landscape in the surrounding patches was a significant predictor for three diversity metrics. These findings indicate that restoration activities have the capacity to increase avian diversity in LSR; however, restoration efforts need to be considered from a landscape context to reach levels equivalent to those found in remnant patches.

### 4.1 Bird Assemblages in Remnant LSR

Connected remnant sites had high bird diversity for all metrics examined in this study, highlighting the importance of conserving remaining connected remnant patches of LSR in northern New South Wales. However, most critically, these connected remnant patches had higher rainforest‐dependent species richness than the revegetated sites. Our results suggest that these connected remnant sites possess the structural requirements, particularly mature canopy trees and a range of ecological niches to support a functionally and phylogenetically unique range of bird species (Catterall et al. 2012; Munro et al. 2011; Laidlaw et al. 2011; Moritz et al. 2000; Floyd 1990). Importantly, a range of rainforest‐dependent species were found at both connected remnant sites such as the brown gerygone ( *Gerygone mouki* ) and pale‐yellow robin ( *Tregellasia capito* ), as well as regionally threatened species including the living fossil species, Albert's lyrebird ( *Menura alberti* ), marbled frogmouth ( *Podargus ocellatus* ), sooty owl ( *Tyto tenebricosa* ), superb fruit dove ( *Ptilinopus superbus* ) and wompoo fruit dove ( *Ptilinopus magnificus* ). As many canopy trees within LSR are fruit‐bearing, they attract specialised, canopy‐dwelling frugivorous species (Huang and Catterall 2021), some of which have distinct lineages predating to the supercontinent Gondwana (i.e., paradise riflebird, green catbird; Mitchell et al. 2021).

The fragmented remnant sites investigated in this study produced contrasting results, despite both sites containing undisturbed remnant vegetation within their boundaries. Hayter's Hill West (fragmented remnant site 2) had consistently low values for all diversity metrics, and the avian species composition at this site was distinct from Andrew Johnston's Big Scrub (fragmented remnant site 1). Although both Andrew Johnston's Big Scrub and Hayter's Hill West possess similar structural and functional vegetation characteristics, 28 bird species detected at Andrew Johnston's Big Scrub were not detected at Hayter's Hill West, and Hayter's Hill West shared many generalist woodland species commonly found at the revegetated sites, including the bar‐shouldered dove ( *Geopelia humeralis* ), eastern barn owl ( *Tyto alba* ), black‐faced cuckooshrike ( *Coracina novaehollandiae* ) and rainbow bee‐eater ( *Merops ornatus* ). One possible explanation for the differences found at Hayter's Hill West may be due to the size and location of this remnant patch. This site is located west of Byron Bay and east of a major highway, is isolated from other patches of LSR vegetation, and has extensive cleared land surrounding the patch. Other studies have found that species suited to colonising marginal gradients of cleared vegetation may prevail in such circumstances, while specialised species may not be able to persist or are unable to recolonise these isolated patches (Pavlacky Jr et al. 2015; Doerr et al. 2011; Catterall et al. 2004). In contrast, Andrew Johnston's Big Scrub displayed individual site results with greatest similarity to connected remnant sites; this site had the highest phylogenetic diversity and the third highest Chao2 species richness and rainforest‐dependent species richness. This is likely due to its larger patch size and increased connectivity and proximity to remnant vegetation, relative to that of Hayter's Hill West, therefore promoting the access and movement of bird species to the site.

### 4.2 Bird Assemblages in Revegetated LSR

The old revegetation sites had lower Chao2 species richness than the young revegetation, and in fact, one of the old revegetation sites, Tarra (OR2), had the lowest rainforest‐dependent species richness of any of the sites examined in this study. These findings were surprising as we expected that the young revegetation would have the lowest avian species richness. A range of species that are generally more tolerant of low‐light and high‐humidity environments were detected at the old revegetation sites, including the Australian brush turkey ( *Alectura lathami* ), wonga pigeon ( *Leucosarcia melanoleuca* ), white‐browed scrubwren ( *Sericornis frontalis* ) and mistletoebird ( *Dicaeum hirundinaceum* ). These species more commonly inhabit less structurally complex environments than remnant LSR (BirdLife Australia 2023). The vegetation structure of the old revegetation sites may have been a contributing factor to low diversity estimates, as key structural features may still be absent, thus limiting the functional niches available to species (Freeman et al. 2015). Late successional rainforest trees are often long‐lived and slow to reach maturity and are generally absent in revegetated LSR communities (Shoo et al. 2013; Floyd 1990). This significantly reduces the structural (e.g., nesting trees, dense vine and epiphyte growth) and nutritional (e.g., fruiting rainforest trees) requirements that rainforest‐dependent species rely on (Bradford and Murphy 2019; Floyd 1990). Due to the age of the old revegetation sites (between 25 and 30 years), it is possible that the established vegetation is in a state of transition that is unfavourable to both generalist and rainforest‐dependent species. Therefore, further maturation may be required before avian assemblages begin to resemble those of remnant LSR patches.

The young revegetation had relatively high Chao2 species richness but low to mid rainforest‐dependent species diversity, functional diversity and phylogenetic diversity when compared to the remnant sites. These sites were characterised by a low proportion of canopy cover, shorter canopy and mid‐story height and prevalent non‐native vegetation benefitting woodland generalist species. For example, the eastern spinebill ( *Acanthorhynchus tenuirostris* ), red‐browed finch ( *Neochmia temporalis* ), eastern barn owl ( *Tyto alba* ) and bar‐shouldered dove ( *Geopelia humeralis* ) are more tolerant of these conditions (BirdLife Australia 2023; Catterall et al. 2012; Freeman et al. 2009). Furthermore, the early developmental stage of the young revegetation may present different functional niches for bird species to utilise (i.e., grass‐dominated understories, aerial hunting opportunities, easier movement through the vegetation mid‐story), which may favour species like the grey goshawk ( *Accipiter novaehollandiae* ) and pheasant coucal ( *Centropus phasianinus* ), which were unique to young remnant sites. Other studies have reported that the presence of a more diverse range of functional niches in young revegetation can lead to increased avian diversity (Batisteli et al. 2018; Freeman et al. 2015). The presence of many generalist bird species at the young revegetation sites led to a reduced cophenetic distance and phylogenetic diversity, as these species have genetically diversified over a short timescale. Furthermore, species that were noted to have ancient ties to LSR vegetation were absent from young revegetation sites, further reducing the cophenetic distance (Mitchell et al. 2021; Jetz et al. 2012). These findings indicate that although young revegetation may have high species richness, these areas have few specialised, phylogenetically distinct species, possibly because structural requirements such as tall canopy and dense vegetation cover may be needed for their presence.

### 4.3 Importance of Vegetation and Landscape Structure

A range of site‐based vegetation attributes was measured in this study; however, these had poor predictive power for trends in species richness and functional diversity. This may be because 57 of the bird species detected in this study were not rainforest‐dependent and occur in multiple ecosystems, thus reducing the efficacy of the models in determining significant predictors (Lelli et al. 2019). However, percentage canopy cover was a near‐significant predictor for rainforest‐dependent species richness. Leach et al. (2018) found a similar relationship between canopy cover and rainforest‐dependent species when exploring the predicted species abundance of montane rainforest birds in south‐east Queensland. High canopy cover provides a low‐light and high‐humidity environment favouring the adaptations of rainforest‐dependent species, particularly ground and subcanopy specialist birds which utilise this dense vegetation and deep humus layers for nutrition, nesting and protection from predation (Palmer and Catterall 2021; Pavlacky Jr et al. 2015; Hagger et al. 2013; Moran et al. 2004; Holmes 1987). Sites with fewer rainforest‐dependent species (i.e., fragmented remnant site 2 and old revegetation site 1) also generally had lower phylogenetic diversity values, and phylogenetically distinct species (such as the living fossil Albert's lyrebird, *Menura alberti* ) were absent from these sites (Mitchell et al. 2021; Sibley and Ahlquist 1985).

While site‐based vegetation attributes were generally not strong predictors of avian diversity, the percentage of vegetated and cleared landscape (i.e., open pastures) within 200 and 500 m of LSR patches showed strong significant relationships with three diversity metrics (species richness, rainforest‐dependent species richness and functional diversity). Similar results have been observed in other studies, where reductions in species richness, abundance and functional diversity of avian communities can occur as the extent of habitat fragmentation increases (Basile et al. 2021; Huang and Catterall 2021; Bregman et al. 2016; Moran and Catterall 2014). Furthermore, as the avian assemblages identified in the current study were comprised of many rainforest‐dependent insectivorous and frugivorous species, losses in LSR and similar remnant vegetation cover can decrease the availability of nutritional sources for species, resulting in their decline or extirpation (Bregman et al. 2016; Moran and Catterall 2014). Conversely, the presence of carnivorous raptors may also be influenced by cleared landscape, as they may roost and nest along margins of LSR vegetation while utilising open environments to prey upon ground‐dwelling species (Mooney 1998). These results indicate that landscape level vegetation coverage may increase diversity by providing connectivity and a larger number of resources. Moreover, the results of this study suggest that sites which are classed as old revegetation may struggle to recoup lost species if they remain fragmented and isolated from other patches (as indicated by low Chao2 species richness and rainforest‐dependent species richness values at old revegetated site 2 –Tarra).

### 4.4 Recommendations for Rainforest Restoration Initiatives

A key finding of this study was that retaining and reconnecting remnant LSR vegetation is critical for the persistence of rainforest‐dependent birds in the landscape. However, this study also revealed that revegetated sites have the capacity to provide habitat for a diversity of avian species. In particular, the young revegetation sites shared many species in common with connected remnant sites, and while this could be due to the proximity of these young revegetated sites to nearby protected areas (i.e., Nightcap National Park), our findings indicate that these sites may provide important resources or refuge in a highly cleared landscape mosaic. These results support the findings of previous literature, which advocates the need to continue and expand upon ecological restoration practices to improve both the recovery and persistence of biodiversity occurring within a broader geographic area (Hagger et al. 2017; Shoo et al. 2013; Parkes et al. 2012; Kanowski et al. 2009).

Three primary actions are therefore recommended for rainforest restoration initiatives. First, remnant vegetation patches should continue to be prioritised for protection, with restoration activities primarily focusing on expanding and reconnecting remnant patches to facilitate the recolonisation and movement of rainforest‐dependent species. Second, restoration activities should seek to establish dense canopy cover (> 90%) across sites to increase the presence of rainforest specialist species. Given the diversity of frugivorous rainforest‐dependent birds found across sites, the establishment of a diverse, fruit‐bearing canopy is highly recommended to retain and/or reintroduce food sources for these species, as well as to promote the passive expansion of rainforest coverage and diversity (Guidetti et al. 2016; Powell et al. 2015; Green 1993). Finally, the strong relationship between three biodiversity metrics and the percentage of vegetation surrounding LSR patches (within a 200 and 500 m radius) reinforces the importance of landscape context and connectivity for LSR avian assemblages.

### 4.5 Limitations and Future Directions

While this study provides valuable insight into avian diversity in LSR, it has some limitations. Firstly, the study was limited in the number of sites surveyed, with only two available per site category. This was unavoidable due to the limited remaining extent of this critically endangered vegetation community, particularly highly connected remnant patches. The low sample size led to a lack of power to detect significant differences in diversity metrics between site categories. Furthermore, the interactions between landscape metrics (such as area and connectivity) and site‐based vegetation attributes could not be modelled. These interactions are likely important for the dispersal and establishment of rainforest species in habitat patches. Secondly, due to logistical constraints, the study only ran over one season (March–June 2024), and thus summer migrants which utilise this landscape would have been largely absent. Species assemblages are known to change seasonally in LSR, and thus patterns in diversity may change in different seasons (Holmes 1987). It is therefore recommended that future studies be repeated in different seasons. Additionally, there were extended periods of rain during the recording period. This meant the number of days which could be analysed was limited as detectability of birds decreases considerably during rain (Ross et al. 2023; Sánchez‐Giraldo et al. 2020). Finally, this study did not compare species diversity in non‐LSR vegetation communities in the landscape. As this study found strong relationships between diversity metrics and landscape connectivity, future work should investigate the role that non‐LSR vegetation plays in facilitating avian dispersal and recolonisation of forest patches in eastern Australia.

## 5 Conclusions

The vegetation of LSR supports a high diversity of bird species in both remnant and revegetated patches. While the findings of this study cannot understate the importance of highly connected remnant vegetation to rainforest avifauna, they also highlight the value of ecological restoration practices for the recovery of avian assemblages in Australian LSR. While no site‐based vegetation attributes were significant predictors of avian diversity, high canopy cover had a strong positive relationship with rainforest‐dependent species. Importantly, the proportion of vegetated land surrounding LSR was found to be a significant positive determinant of avian biodiversity for three of the metrics examined. Future restoration efforts should aim to establish mature canopy cover in LSR ecosystems, to increase the presence of rainforest‐dependent species and restore the connectivity and extent of LSR vegetation for avian assemblages to utilise. Applying these practices will allow for the persistence of LSR avian communities indefinitely, in turn retaining the important ecological functions they provide to this critically endangered ecosystem.

## Author Contributions

**Marley Borrow:** conceptualization (equal), data curation (equal), formal analysis (equal), investigation (equal), methodology (equal), validation (equal), visualization (equal), writing – original draft (equal), writing – review and editing (equal). **Susan Fuller:** conceptualization (equal), methodology (equal), project administration (lead), supervision (equal), writing – original draft (equal), writing – review and editing (equal). **Brendan Doohan:** data curation (equal), formal analysis (equal), investigation (equal), methodology (equal), supervision (equal), validation (equal), visualization (equal), writing – original draft (equal), writing – review and editing (equal).

## Ethics Statement

This research was conducted under the following animal ethics and scientific research permits: New South Wales Secretary's Animal Care and Ethics Committee project number 17/979, Big Scrub Rainforest Conservancy scientific licence SL102488 and Queensland University of Technology administrative approval project 8910.

## Conflicts of Interest

The authors declare no conflicts of interest.

## Supporting information


**Appendix S1:** ece372345‐sup‐0001‐Appendix.docx.

## Data Availability

The data that support the findings of this study are openly available in figshare at https://doi.org/10.6084/m9.figshare.29043689.
