# Supplementary material for: Rebound From Restoration: Assessing the Diversity of Bird Assemblages in Revegetated and Remnant Patches of Critically Endangered Lowland Subtropical Rainforest
Source: Ecol Evol. 2025 Oct 17;15(10):e72345. doi: 10.1002/ece3.72345 (PMC12531714; doi:10.1002/ece3.72345)
Supplement: Supplementary file 1 — Appendix S1: ece372345‐sup‐0001‐Appendix.docx. [file ECE3-15-e72345-s001.docx]

**Appendices**

*Appendix A: Phylogenetic tree created using the Jetz et al., 2014 dataset.*


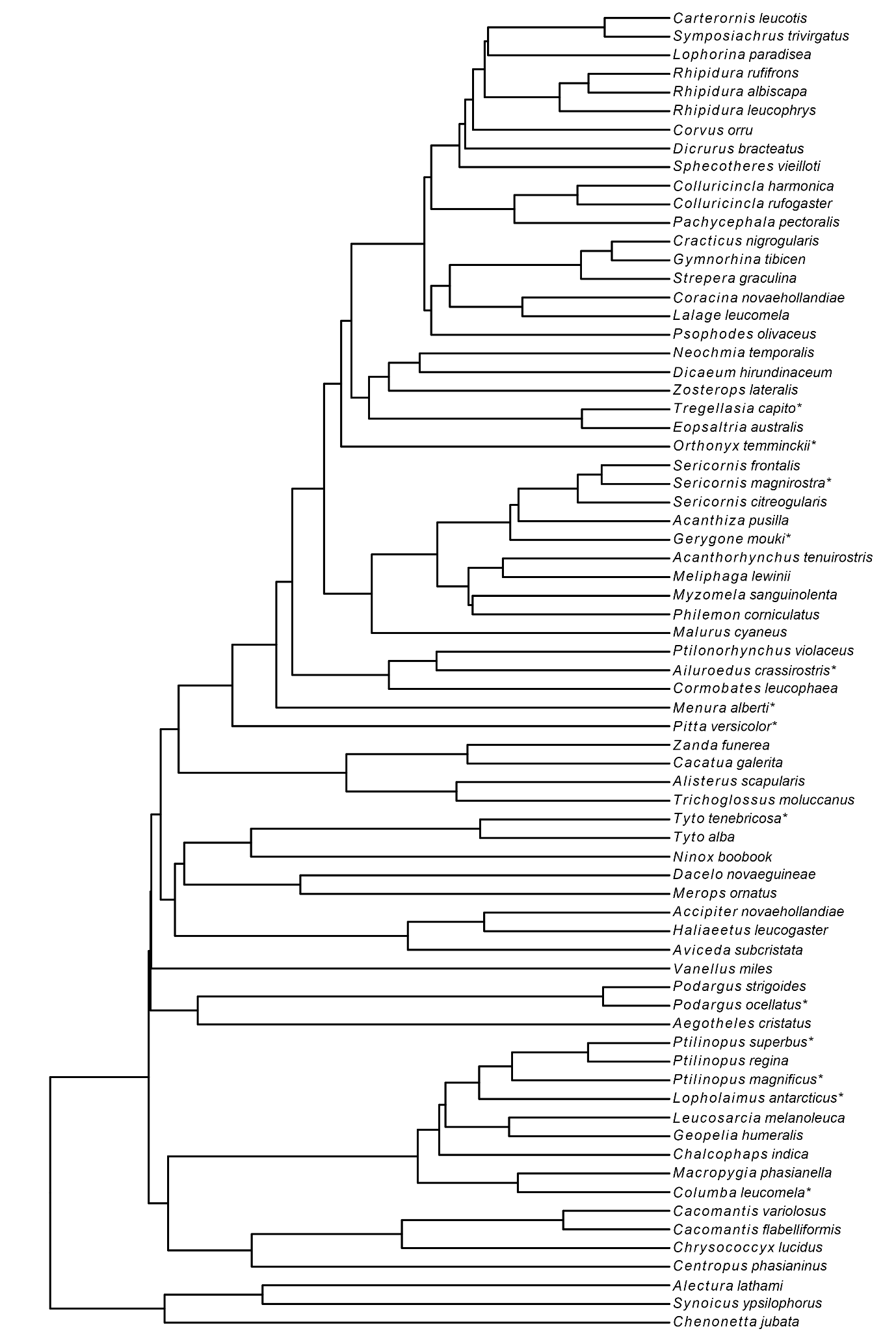


Phylogenetic tree for all sites combined. Asterisks (*) indicate rainforest dependent species.

*Appendix B: Species presence-absence matrix and species richness for each site.*

Presence-absence matrix of the species identified across sites. Species presence = 1, absence = 0. Categories: CR = Connected Remnant, FR = Fragmented Remnant, OR = Old Revegetation, YR = Young Revegetation. Rainforest-dependent species are indicated with an asterisk (*).

| **Scientific Name** | **Common Name** | **CR1** | [**CR2**](rewritten://1c2a3492-a922-44e7-b410-27247108757e) | **FR1** | **FR2** | **OR1** | **OR2** | **YR1** | **YR2** |
| --- | --- | --- | --- | --- | --- | --- | --- | --- | --- |
| *Menura alberti** | Albert's Lyrebird | 0 | 1 | 0 | 0 | 0 | 0 | 0 | 0 |
| *Alectura lathami* | Australian Brush turkey | 1 | 1 | 0 | 0 | 1 | 1 | 0 | 0 |
| [*Sphecotheres vieilloti*](https://www.birdsinbackyards.net/Passeriformes/Oriolidae/Sphecotheres/Sphecotheres-vieilloti) | Australian Figbird | 1 | 1 | 1 | 1 | 1 | 1 | 1 | 1 |
| *Alisterus scapularis* | Australian King Parrot | 0 | 1 | 0 | 0 | 0 | 0 | 1 | 0 |
| *Orthonyx temminckii** | Australian Logrunner | 1 | 1 | 1 | 1 | 1 | 1 | 1 | 1 |
| *Gymnorhina tibicen* | Australian Magpie | 1 | 1 | 1 | 1 | 1 | 0 | 1 | 1 |
| *Aegotheles cristatus* | Australian Owlet-nightjar | 1 | 0 | 1 | 0 | 0 | 0 | 0 | 0 |
| *Chenonetta jubata* | Australian Wood Duck | 0 | 1 | 1 | 0 | 1 | 0 | 1 | 0 |
| *Geopelia humeralis* | Bar-shouldered Dove | 0 | 0 | 0 | 1 | 1 | 1 | 1 | 1 |
| *Tyto alba* | Eastern Barn Owl | 0 | 0 | 0 | 1 | 1 | 1 | 1 | 0 |
| *Coracina novaehollandiae* | Black-faced Cuckooshrike | 0 | 0 | 0 | 1 | 1 | 0 | 1 | 0 |
| *Chalcophaps longirostris** | Brown-capped Emerald Dove | 1 | 1 | 1 | 0 | 1 | 1 | 1 | 1 |
| *Macropygia phasianella* | Brown Cuckoo-dove | 1 | 1 | 1 | 1 | 1 | 1 | 1 | 1 |
| *Gerygone mouki** | Brown Gerygone | 1 | 1 | 1 | 0 | 0 | 0 | 0 | 0 |
| *Synoicus ypsilophorus* | Brown Quail | 0 | 0 | 0 | 1 | 0 | 0 | 0 | 0 |
| *Acanthiza pusilla* | Brown Thornbill | 1 | 1 | 1 | 0 | 1 | 1 | 1 | 1 |
| *Cacomantis variolosus* | Brush Cuckoo | 1 | 0 | 1 | 0 | 0 | 0 | 0 | 1 |
| *Acanthorhynchus tenuirostris* | Eastern Spinebill | 0 | 0 | 0 | 0 | 0 | 0 | 1 | 0 |
| *Psophodes olivaceus* | Eastern Whipbird | 1 | 1 | 1 | 1 | 1 | 1 | 1 | 1 |
| Eopsaltria australis | Eastern Yellow Robin | 1 | 1 | 1 | 1 | 1 | 1 | 1 | 1 |
| *Cacomantis flabelliformis* | Fan-tailed Cuckoo | 1 | 1 | 1 | 1 | 1 | 1 | 1 | 1 |
| *Pachycephala pectoralis* | Golden Whistler | 1 | 1 | 1 | 1 | 1 | 1 | 1 | 1 |
| *Ailuroedus crassirostris** | Green Catbird | 1 | 1 | 1 | 1 | 1 | 1 | 1 | 1 |
| *Rhipidura albiscapa* | Grey Fantail | 1 | 1 | 1 | 1 | 1 | 1 | 1 | 1 |
| *Accipiter novaehollandiae* | Grey Goshawk | 0 | 0 | 0 | 0 | 0 | 0 | 1 | 0 |
| *Colluricincla harmonica* | Grey Shrike-thrush | 1 | 1 | 1 | 1 | 1 | 1 | 1 | 1 |
| *Sericornis magnirostra** | Large-billed Scrubwren | 1 | 1 | 1 | 1 | 1 | 1 | 1 | 1 |
| *Dacelo novaeguineae* | Laughing Kookaburra | 1 | 1 | 1 | 1 | 1 | 1 | 1 | 1 |
| *Meliphaga lewinii* | Lewin’s Honeyeater | 1 | 1 | 1 | 1 | 1 | 1 | 1 | 1 |
| *Colluricincla rufogaster* | Rufous Shrikethrush | 1 | 1 | 1 | 1 | 1 | 1 | 1 | 1 |
| *Podargus ocellatus** | Marbled Frogmouth | 0 | 1 | 0 | 0 | 0 | 0 | 0 | 0 |
| *Vanellus miles* | Masked Lapwing | 1 | 1 | 1 | 1 | 1 | 1 | 0 | 1 |
| *Dicaeum hirundinaceum* | Mistletoebird | 1 | 1 | 1 | 1 | 1 | 1 | 1 | 1 |
| *Philemon corniculatus* | Noisy Friarbird | 1 | 1 | 0 | 0 | 0 | 0 | 1 | 0 |
| *Pitta versicolor** | Noisy Pitta | 1 | 1 | 1 | 1 | 1 | 1 | 1 | 1 |
| *Aviceda subcristata* | Pacific Baza | 0 | 0 | 0 | 0 | 0 | 0 | 0 | 1 |
| *Tregellasia capito** | Pale-yellow Robin | 1 | 1 | 1 | 0 | 0 | 0 | 0 | 0 |
| *Lophorina paradisea** | Paradise Riflebird | 1 | 1 | 0 | 0 | 0 | 0 | 0 | 0 |
| *Centropus phasianinus* | Pheasant Coucal | 0 | 0 | 0 | 0 | 0 | 0 | 0 | 1 |
| *Cracticus nigrogularis* | Pied Butcherbird | 0 | 1 | 0 | 1 | 1 | 1 | 1 | 1 |
| *Strepera graculina* | Pied Currawong | 1 | 1 | 1 | 1 | 1 | 1 | 1 | 1 |
| *Merops ornatus* | Rainbow Bee-eater | 0 | 0 | 0 | 1 | 0 | 0 | 0 | 1 |
| *Trichoglossus moluccanus* | Rainbow Lorikeet | 1 | 1 | 1 | 1 | 1 | 1 | 1 | 1 |
| *Neochmia temporalis* | Red-browed Finch | 0 | 0 | 0 | 1 | 0 | 1 | 1 | 1 |
| *Ptilinopus regina* | Rose-crowned Fruit Dove | 1 | 1 | 1 | 1 | 1 | 1 | 1 | 1 |
| *Rhipidura rufifrons* | Australian Rufous Fantail | 1 | 1 | 1 | 1 | 1 | 0 | 1 | 1 |
| *Ptilonorhynchus violaceus* | Satin Bowerbird | 0 | 1 | 1 | 0 | 1 | 0 | 1 | 1 |
| *Myzomela sanguinolenta* | Scarlet Myzolema | 1 | 1 | 0 | 0 | 0 | 1 | 1 | 1 |
| *Chrysococcyx lucidus* | Shining Bronze-cuckoo | 1 | 1 | 1 | 1 | 1 | 1 | 1 | 1 |
| *Zosterops lateralis* | Silvereye | 1 | 1 | 1 | 1 | 1 | 1 | 1 | 1 |
| *Tyto tenebricosa** | Sooty Owl | 0 | 1 | 1 | 0 | 0 | 0 | 0 | 1 |
| *Ninox boobook* | Southern Boobook | 1 | 1 | 1 | 0 | 1 | 0 | 0 | 1 |
| *Dicrurus bracteatus* | Spangled Dongo | 1 | 1 | 1 | 1 | 1 | 1 | 1 | 1 |
| *Symposiachrus trivirgatus* | Spectacled Monarch | 1 | 1 | 0 | 1 | 0 | 0 | 1 | 1 |
| *Cacatua galerita* | Sulphur-crested Cockatoo | 1 | 1 | 1 | 0 | 1 | 0 | 0 | 1 |
| *Malurus cyaneus* | Superb Fairywren | 1 | 1 | 0 | 0 | 1 | 0 | 0 | 1 |
| *Ptilinopus superbus** | Superb Fruit Dove | 1 | 1 | 0 | 0 | 0 | 0 | 0 | 0 |
| *Podargus strigoides* | Tawny Frogmouth | 0 | 0 | 1 | 0 | 0 | 0 | 0 | 1 |
| *Lopholaimus antarcticus** | Topknot Pigeon | 1 | 1 | 1 | 0 | 1 | 0 | 1 | 1 |
| *Corvus orru* | Torresian Crow | 1 | 1 | 0 | 1 | 1 | 1 | 1 | 1 |
| *Lalage leucomela* | Varied Triller | 1 | 1 | 1 | 1 | 1 | 1 | 1 | 1 |
| *Haliaeetus leucogaster* | White-bellied Sea Eagle | 1 | 0 | 1 | 0 | 0 | 0 | 0 | 0 |
| *Sericornis frontalis* | White-browed Scrubwren | 1 | 1 | 1 | 0 | 1 | 1 | 1 | 1 |
| *Carterornis leucotis* | White-eared Monarch | 0 | 0 | 0 | 1 | 0 | 0 | 1 | 1 |
| *Columba leucomela** | White=headed Pigeon | 1 | 1 | 1 | 1 | 1 | 0 | 1 | 1 |
| *Cormobates leucophaea* | White-throated Treecreeper | 1 | 1 | 1 | 0 | 0 | 0 | 1 | 0 |
| *Rhipidura leucophrys* | Willie Wagtail | 0 | 0 | 0 | 0 | 1 | 1 | 0 | 0 |
| *Ptilinopus magnificus** | Wompoo Fruit Dove | 1 | 1 | 1 | 1 | 0 | 0 | 0 | 0 |
| *Leucosarcia melanoleuca* | Wonga Pigeon | 1 | 1 | 1 | 0 | 1 | 1 | 1 | 1 |
| *Zanda funerea* | Yellow-tailed Black Cockatoo | 1 | 1 | 0 | 0 | 0 | 1 | 1 | 1 |
| *Sericornis citreogularis* | Yellow-throated Scrubwren | 1 | 1 | 1 | 0 | 1 | 0 | 0 | 1 |
| **Species Richness** | | 52 | 55 | 46 | 38 | 45 | 37 | 48 | 51 |
| **Rainforest-dependent Species Richness** | | 12 | 15 | 11 | 6 | 7 | 5 | 7 | 8 |

*Appendix C: Functional diversity matrix*

Functional attributes used to calculate Functional Diversity (FD) across sites. Stratum layers denote 1= Ground, 2= Mid Story, 3 = Canopy.

| Common Name | Feeding Group | Stratum | Foraging Activity | Body Mass |
| --- | --- | --- | --- | --- |
| Albert's Lyrebird | Insectivore | 1 | Diurnal | 928 |
| Australian Brushturkey | Herbivore | 1 | Diurnal | 2333.06 |
| Australian Figbird | Frugivore | 3 | Diurnal | 108 |
| Australian King Parrot | Herbivore | 3 | Diurnal | 232.26 |
| Australian Logrunner | Insectivore | 1 | Diurnal | 58.06 |
| Australian Magpie | Insectivore | 1 | Diurnal | 284.87 |
| Australian Owlet-nightjar | Insectivore | 2 | Nocturnal | 42.78 |
| Australian Wood Duck | Herbivore | 1 | Diurnal | 807.46 |
| Bar-shouldered Dove | Granivore | 1 | Diurnal | 128.42 |
| Eastern Barn Owl | Carnivore | 1 | Nocturnal | 403.32 |
| Black-faced Cuckooshrike | Insectivore | 3 | Diurnal | 118 |
| Brown-capped Emerald Dove | Frugivore | 1 | Diurnal | 136.54 |
| Brown Cuckoo-dove | Frugivore | 3 | Diurnal | 237 |
| Brown Gerygone | Insectivore | 2 | Diurnal | 5.2 |
| Brown Quail | Granivore | 1 | Diurnal | 107.69 |
| Brown Thornbill | Insectivore | 2 | Diurnal | 7.62 |
| Brush Cuckoo | Insectivore | 3 | Diurnal | 35.75 |
| Eastern Spinebill | Nectarivore | 2 | Diurnal | 10.44 |
| Eastern Yellow Robin | Insectivore | 2 | Diurnal | 20.15 |
| Eastern Whipbird | Insectivore | 2 | Diurnal | 62.36 |
| Fan-tailed Cuckoo | Insectivore | 2 | Diurnal | 49.8 |
| Golden Whistler | Insectivore | 2 | Diurnal | 30.36 |
| Green Catbird | Frugivore | 3 | Diurnal | 205.32 |
| Grey Fantail | Insectivore | 2 | Diurnal | 7.53 |
| Grey Goshawk | Carnivore | 3 | Diurnal | 326.06 |
| Grey Shrikethrush | Carnivore | 3 | Diurnal | 65.8 |
| Large-billed Scrubwren | Insectivore | 1 | Diurnal | 9.8 |
| Laughing Kookaburra | Carnivore | 3 | Diurnal | 333.8 |
| Lewin’s Honeyeater | Frugivore | 3 | Diurnal | 33.58 |
| Rufous Shrikethrush | Insectivore | 2 | Diurnal | 32.34 |
| Marbled Frogmouth | Insectivore | 3 | Nocturnal | 175.37 |
| Masked Lapwing | Insectivore | 1 | Diurnal | 387 |
| Mistletoebird | Frugivore | 3 | Diurnal | 9.04 |
| Noisy Friarbird | Nectarivore | 3 | Diurnal | 100.71 |
| Noisy Pitta | Insectivore | 1 | Diurnal | 88.94 |
| Pacific Baza | Insectivore | 3 | Diurnal | 322.65 |
| Pale-yellow Robin | Insectivore | 2 | Diurnal | 13.9 |
| Paradise Riflebird | Frugivore | 3 | Diurnal | 119.49 |
| Pheasant Coucal | Carnivore | 1 | Diurnal | 381.98 |
| Pied Butcherbird | Insectivore | 3 | Diurnal | 128 |
| Pied Currawong | Herbivore | 3 | Diurnal | 298.86 |
| Rainbow Bee-eater | Insectivore | 3 | Diurnal | 29.5 |
| Rainbow Lorikeet | Nectarivore | 3 | Diurnal | 113.25 |
| Red-browed Finch | Granivore | 1 | Diurnal | 11.4 |
| Rose-crowned Fruit Dove | Frugivore | 3 | Diurnal | 101.57 |
| Rufous Fantail | Insectivore | 2 | Diurnal | 10.08 |
| Satin Bowerbird | Frugivore | 3 | Diurnal | 218.91 |
| Scarlet Myzolema | Nectarivore | 3 | Diurnal | 8.34 |
| Shining Bronze-cuckoo | Insectivore | 3 | Diurnal | 24.44 |
| Silvereye | Frugivore | 2 | Diurnal | 12.68 |
| Sooty Owl | Carnivore | 3 | Nocturnal | 660.6 |
| Southern Boobook | Carnivore | 3 | Nocturnal | 282.19 |
| Spangled Dongo | Insectivore | 3 | Diurnal | 85.53 |
| Spectacled Monarch | Insectivore | 2 | Diurnal | 12.7 |
| Sulphur-crested Cockatoo | Herbivore | 3 | Diurnal | 720.43 |
| Superb Fairywren | Insectivore | 2 | Diurnal | 10.33 |
| Superb Fruit Dove | Frugivore | 3 | Diurnal | 118 |
| Tawny Frogmouth | Carnivore | 1 | Nocturnal | 308.03 |
| Topknot Pigeon | Frugivore | 3 | Diurnal | 496.37 |
| Torresian Crow | Granivore | 3 | Diurnal | 507.96 |
| Varied Triller | Frugivore | 3 | Diurnal | 29.25 |
| White-bellied Sea Eagle | Carnivore | 1 | Diurnal | 2827.01 |
| White-browed Scrubwren | Insectivore | 1 | Diurnal | 13.23 |
| White-eared Monarch | Insectivore | 3 | Diurnal | 11.4 |
| White-headed Pigeon | Frugivore | 3 | Diurnal | 420 |
| White-throated Treecreeper | Insectivore | 3 | Diurnal | 22 |
| Willie Wagtail | Insectivore | 2 | Diurnal | 27.44 |
| Wompoo Fruit Dove | Frugivore | 3 | Diurnal | 185.41 |
| Wonga Pigeon | Granivore | 1 | Diurnal | 428.57 |
| Yellow-tailed Black Cockatoo | Granivore | 3 | Diurnal | 674.72 |
| Yellow-throated Scrubwren | Insectivore | 1 | Diurnal | 17.6 |

*Appendix D: Summary of vegetation data by site category.*

Summary statistics (mean and standard deviation) of vegetation metrics

| Site Category | Canopy Cover | Mid Story Cover | Canopy Height | Upper Mid Canopy Height | Lower Mid Canopy Height | Canopy Width | Upper Mid Story Width | Lower Mid Story Width | Woody Debris Length | Organic Litter Cover |
| --- | --- | --- | --- | --- | --- | --- | --- | --- | --- | --- |
| Connected Remnant | 0.92 (0.03) | 0.74 (0.08) | 21.05 (0.07) | 7.5 (0.71) | 3.45 (0.21) | 37.7 (7.21) | 6.41 (1.07) | 2.05 (0.23) | 215.88 (150.08) | 0.58 (0.26) |
| Fragmented Remnant | 0.85 (0.04) | 0.83 (0.03) | 22.02 (6.26) | 8.53 (1.1) | 3.87 (0.41) | 38.4 (15.91) | 6.81 (0.98) | 2.72 (0.11) | 157.72 (47.38) | 0.42 (0.28) |
| Old Revegetation | 0.85 (0.01) | 0.67 (0.28) | 18.6 (3.18) | 7.2 (0.31) | 3.22 (0.74) | 27.93 (12.13) | 8.28 (0.25) | 3.2 (0.99) | 164.94 (37.33) | 0.53 (0.1) |
| Young Revegetation | 0.77 (0.01) | 0.91 (0.07) | 17.91 (0.22) | 8.39 (1.57) | 2.78 (0.04) | 19.5 (2.43) | 4.98 (0.48) | 2.35 (1.34) | 228.55 (196.5) | 0.5 (0.2) |

*
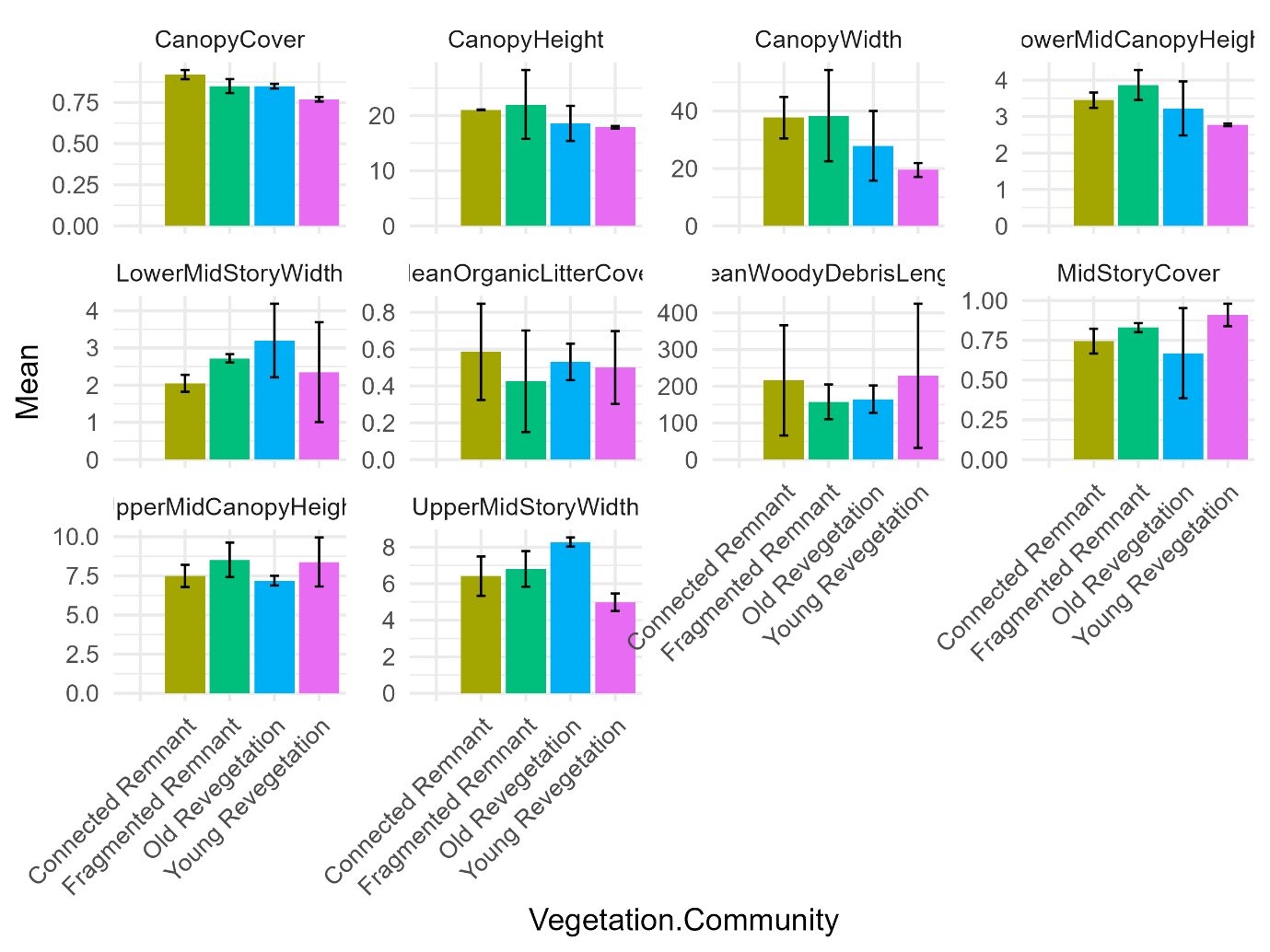
*

*Figure D.1.* Graph of vegetation characteristics by site category.

*Appendix E: Relationship between diversity metrics and site-based vegetation and landscape attributes*

Regression models for Chao2 species richness, rainforest-dependent species richness, functional diversity and phylogenetic diversity with study site vegetation attributes and landscapes attributes. Slopes and permutation p values were generated after 9,999 resamplings of survey data. Confidence intervales (CI) were generated using 1000 resampling bootstraps.

| Attribute | Response | Predictor | Slope (95% CI) | p (perm) |
| --- | --- | --- | --- | --- |
| Vegetation | Chao 2 Species richness | Canopy Cover (%) | 21.45 (-98.55, 186.17) | 0.703 |
|  |  | Mid Story Cover (%) | 7.9 (-101.54, 28.93) | 0.739 |
|  |  | Canopy Height (m) | -0.87 (-2.4, 2.96) | 0.414 |
|  |  | Upper Mid Story Height (m) | 1.76 (-11.38, 7.35) | 0.612 |
|  |  | Lower Mid Story Height (m) | 2.25 (-10.45, 14.95) | 0.713 |
|  |  | Canopy Width (cm) | -0.25 (-0.66, 0.67) | 0.393 |
|  |  | Upper Mid Story Width (cm) | -3.27 (-7.21, 0.38) | 0.152 |
|  |  | Lower Mid Story Width (cm) | -3.6 (-14.04, 3.64) | 0.406 |
|  |  | Mean Woody Debris Length (cm) | 0.04 (-0.12, 0.09) | 0.194 |
|  |  | Mean Organic Litter Cover (%) | -14.41 (-52.43, 43) | 0.443 |
|  | Rainforest-dependent species richness | Canopy Cover (%) | 37.87 (-7.33, 106.73) | 0.080 |
|  |  | Mid Story Cover (%) | 2.74 (-54.49, 13.96) | 0.787 |
|  |  | Canopy Height (m) | 0.06 (-0.53, 1.92) | 0.901 |
|  |  | Upper Mid Story Height (m) | 0.36 (-4.27, 2.97) | 0.819 |
|  |  | Lower Mid Story Height (m) | 1.82 (-2.52, 6.42) | 0.495 |
|  |  | Canopy Width (cm) | 0.06 (-0.19, 0.53) | 0.648 |
|  |  | Upper Mid Story Width (cm) | -0.39 (-3.08, 1.71) | 0.710 |
|  |  | Lower Mid Story Width (cm) | -1.96 (-5.32, 1.55) | 0.259 |
|  |  | Mean Woody Debris Length (cm) | 0.01 (-0.02, 0.04) | 0.328 |
|  |  | Mean Organic Litter Cover (%) | -5.18 (-24.92, 10.72) | 0.519 |
|  | Functional diversity | Canopy Cover (%) | 2.74 (0.26, 9.42) | 0.265 |
|  |  | Mid Story Cover (%) | 0.36 (-2.99, 1.52) | 0.703 |
| Vegetation |  | Canopy Height (m) | -0.05 (-0.13, 0.12) | 0.251 |
|  |  | Upper Mid Story Height (m) | 0.06 (-0.5, 0.29) | 0.714 |
|  |  | Lower Mid Story Height (m) | -0.03 (-0.75, 0.34) | 0.93 |
|  |  | Canopy Width (cm) | -0.01 (-0.04, 0.03) | 0.531 |
|  |  | Upper Mid Story Width (cm) | 0.05 (-0.12, 0.29) | 0.648 |
|  |  | Lower Mid Story Width (cm) | -0.13 (-0.43, 0.24) | 0.516 |
|  |  | Mean Woody Debris Length (cm) | 0 (0, 0.01) | 0.365 |
|  |  | Mean Organic Litter Cover (%) | -1.08 (-3.06, 0.06) | 0.21 |
|  | Phylogenetic diversity | Canopy Cover (%) | 36 (4.76, 102.46) | 0.15 |
|  |  | Mid Story Cover (%) | -7.24 (-50.08, 2.4) | 0.512 |
|  |  | Canopy Height (m) | -0.54 (-1.21, 0.76) | 0.266 |
|  |  | Upper Mid Story Height (m) | 1.55 (-4.68, 5.05) | 0.334 |
|  |  | Lower Mid Story Height (m) | 4.33 (-1.41, 7.84) | 0.121 |
|  |  | Canopy Width (cm) | -0.06 (-0.35, 0.34) | 0.681 |
|  |  | Upper Mid Story Width (cm) | 0.99 (-1.19, 3.26) | 0.377 |
|  |  | Lower Mid Story Width (cm) | 1.44 (-1.89, 4.94) | 0.481 |
|  |  | Mean Woody Debris Length (cm) | 0.01 (-0.01, 0.09) | 0.329 |
|  |  | Mean Organic Litter Cover (%) | -11.86 (-28.35, 5.2) | 0.158 |
| Landscape | Species richness | Site Area (ha) | 0.05 (-0.03, 0.69) | 0.402 |
|  |  | % Vegetation vs Cleared (100m buffer) | 22.94 (-521.14, 67.65) | 0.582 |
|  |  | % LSR vs Other Vegetation (100m buffer) | -6.13 (-22.87, 30.13) | 0.593 |
|  |  | % Vegetated Land (200m buffer) | 36.22 (22.08, 65.35) | **0.0084*** |
|  |  | % Native vs Non-Native Vegetation (200m buffer) | -104.76 (-884.76, 75.59) | 0.479 |
|  |  | % LSR vs Other Vegetation (200m buffer) | 12.57 (-19.76, 37.47) | 0.4 |
|  |  | % Vegetated Land (500m buffer) | 25.58 (10.61, 41.59) | **0.0147*** |
|  |  | % Native vs Non-Native Vegetation (500m buffer) | 14.23 (-38.75, 32.51) | 0.255 |
|  |  | % LSR vs Other Vegetation (500m buffer) | 7.59 (-18.06, 29.57) | 0.507 |
|  | Rainforest-dependent species richness | Site Area (ha) | 0.02 (-0.03, 0.22) | 0.395 |
| Landscape |  | % Vegetation vs Cleared (100m buffer) | 15.45 (-73.37, 313.7) | 0.336 |
|  |  | % LSR vs Other Vegetation (100m buffer) | -2.73 (-10.12, 11.38) | 0.575 |
|  |  | % Vegetated Land (200m buffer) | 13.23 (4.42, 24.28) | **0.0301*** |
|  |  | % Native vs Non-Native Vegetation (200m buffer) | 23.08 (-28.18, 439.43) | 0.909 |
|  |  | % LSR vs Other Vegetation (200m buffer) | 1.89 (-10.84, 13.22) | 0.788 |
|  |  | % Vegetated Land (500m buffer) | 9.5 (2.42, 15.36) | **0.0401*** |
|  |  | % Native vs Non-Native Vegetation (500m buffer) | 6.7 (-1.16, 14.51) | 0.206 |
|  |  | % LSR vs Other Vegetation (500m buffer) | 3.96 (-5.71, 12.63) | 0.416 |
|  | Functional diversity | Site Area (ha) | 0 (0, 0.02) | 0.678 |
|  |  | % Vegetation vs Cleared (100m buffer) | 1.16 (-6.65, 27.76) | 0.509 |
|  |  | % LSR vs Other Vegetation (100m buffer) | -0.32 (-1.33, 0.7) | 0.554 |
|  |  | % Vegetated Land (200m buffer) | 1.42 (-0.35, 2.44) | **0.0336*** |
|  |  | % Native vs Non-Native Vegetation (200m buffer) | 0.61 (-19.19, 27.32) | 0.88 |
|  |  | % LSR vs Other Vegetation (200m buffer) | 0.44 (-0.64, 1.83) | 0.565 |
|  |  | % Vegetated Land (500m buffer) | 0.77 (-0.63, 1.63) | 0.147 |
|  |  | % Native vs Non-Native Vegetation (500m buffer) | -0.25 (-1.53, 0.85) | 0.691 |
|  |  | % LSR vs Other Vegetation (500m buffer) | -0.13 (-1.42, 0.87) | 0.81 |
|  | Phylogenetic diversity | Site Area (ha) | 0 (-0.08, 0.26) | 0.924 |
|  |  | % Vegetation vs Cleared (100m buffer) | -6.46 (-61.27, 629.94) | 0.752 |
|  |  | % LSR vs Other Vegetation (100m buffer) | -3.45 (-10.28, 8.93) | 0.525 |
|  |  | % Vegetated Land (200m buffer) | 9.42 (-5.55, 23.11) | 0.227 |
|  |  | % Native vs Non-Native Vegetation (200m buffer) | 9.38 (-46.35, 773.08) | 0.947 |
|  |  | % LSR vs Other Vegetation (200m buffer) | 2.61 (-9.68, 17.48) | 0.719 |
|  |  | % Vegetated Land (500m buffer) | 3.42 (-8.8, 13.38) | 0.562 |
|  |  | % Native vs Non-Native Vegetation (500m buffer) | 3.81 (-20.37, 15.29) | 0.52 |
|  |  | % LSR vs Other Vegetation (500m buffer) | 3.22 (-5.28, 20.99) | 0.549 |
